# SARS-CoV-2 infection of the pancreas promotes thrombofibrosis and is associated with new-onset diabetes

**DOI:** 10.1172/jci.insight.151551

**Published:** 2021-08-23

**Authors:** Mirza Muhammad Fahd Qadir, Manika Bhondeley, Wandy Beatty, Dina D. Gaupp, Lara A. Doyle-Meyers, Tracy Fischer, Ishitri Bandyopadhyay, Robert V. Blair, Rudolf Bohm, Jay Rappaport, Eric Lazartigues, Richard S. Vander Heide, Jay K. Kolls, Xuebin Qin, Franck Mauvais-Jarvis

**Affiliations:** 1 Section of Endocrinology and Metabolism, Deming Department of Medicine, Tulane University School of Medicine, New Orleans, Louisiana, USA.; 2 Southeast Louisiana Veterans Affairs Healthcare System, New Orleans, Louisiana, USA.; 3 Tulane Center of Excellence in Sex-Based Biology and Medicine, New Orleans, Louisiana, USA.; 4 Molecular Microbiology Imaging facility, Washington University School of Medicine, St. Louis, Missouri, USA.; 5 Center for Stem Cell Research and Regenerative Medicine, Tulane University School of Medicine, New Orleans, Louisiana, USA.; 6 Tulane National Primate Research Center, Covington, Louisiana, USA.; 7 Department of Pharmacology and Experimental Therapeutics and; 8 Cardiovascular Center of Excellence, Louisiana State University Health Sciences Center, New Orleans, Louisiana, USA.; 9 Department of Pathology, Louisiana State University, New Orleans, Louisiana, USA.; 10 Center for Translational Research in Infection and Inflammation, Tulane University School of Medicine, New Orleans, Louisiana, USA.

**Keywords:** COVID-19, Diabetes, Endothelial cells, Thrombosis

## Abstract

Evidence suggests an association between severe acute respiratory syndrome–cornavirus-2 (SARS-CoV-2) infection and the occurrence of new-onset diabetes. We examined pancreatic expression of angiotensin-converting enzyme 2 (ACE2) and transmembrane serine protease 2 (TMPRSS2), the cell entry factors for SARS-CoV-2, using publicly available single-cell RNA sequencing data sets, and pancreatic tissue from control male and female nonhuman primates (NHPs) and humans. We also examined SARS-CoV-2 immunolocalization in pancreatic cells of SARS-CoV-2–infected NHPs and patients who had died from coronavirus disease 2019 (COVID-19). We report expression of ACE2 in pancreatic islet, ductal, and endothelial cells in NHPs and humans. In pancreata from SARS-CoV-2–infected NHPs and COVID-19 patients, SARS-CoV-2 infected ductal, endothelial, and islet cells. These pancreata also exhibited generalized fibrosis associated with multiple vascular thrombi. Two out of 8 NHPs developed new-onset diabetes following SARS-CoV-2 infection. Two out of 5 COVID-19 patients exhibited new-onset diabetes at admission. These results suggest that SARS-CoV-2 infection of the pancreas may promote acute and especially chronic pancreatic dysfunction that could potentially lead to new-onset diabetes.

## Graphical abstract

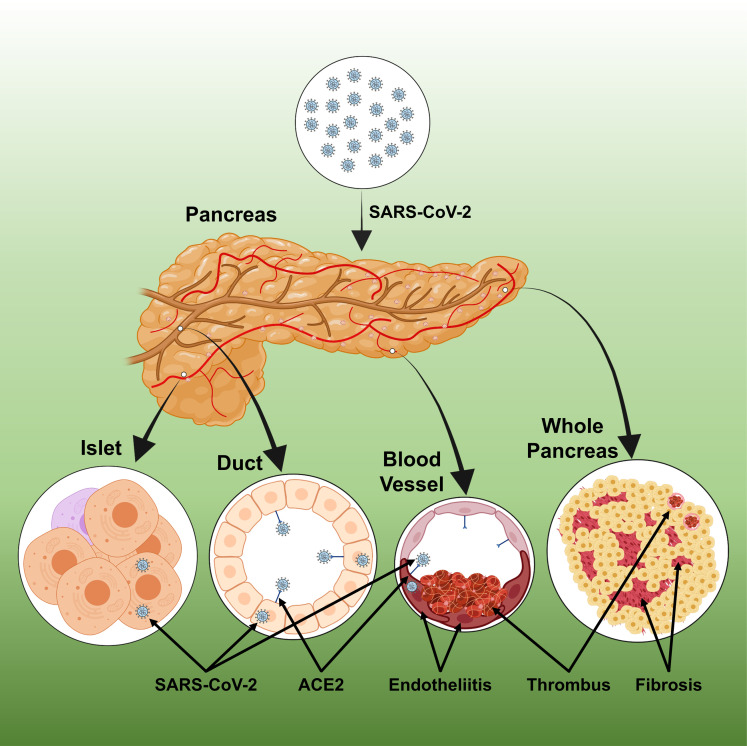

## Introduction

Coronavirus disease 2019 (COVID-19), which is caused by severe acute respiratory syndrome–cornavirus-2 (SARS-CoV-2), affects multiple tissues, including the pancreas (1). Evidence suggests that COVID-19 is associated with new onset of ketosis-prone forms of diabetes or the insulinopenic decompensation of preexisting diabetes (2–4). These observations raise the prospect of a diabetogenic effect of SARS-CoV-2, beyond the known stress response associated with acute illness. For example, SARS-CoV-2 infection may produce new mechanisms of pancreatic insulin-producing β cell failure or aggravate the pathophysiology of β cell dysfunction in preexisting type 2 diabetes (3, 5). In parallel, emerging evidence suggests that COVID-19 produces pancreatic exocrine dysfunction resulting in acute pancreatitis (6). Still, epidemiological data supporting the idea that COVID-19 leads to new onset of insulin-deficient diabetes are limited.

SARS-CoV-2 cellular entry is mediated by angiotensin-converting enzyme 2 (ACE2) and transmembrane serine protease 2 (TMPRSS2), which together anchor and cleave SARS-CoV-2’s spike glycoproteins, allowing for viral internalization (7). Studies on ACE2 expression in pancreatic insulin-producing β cells have shown conflicting results, with some studies reporting ACE2 expression in β cells (8–11) and others showing little or no expression (12, 13). Nevertheless, multiple studies report the expression of ACE2 and TMPRSS2 in pancreatic exocrine ductal cells (8, 9, 12, 13).

SARS-CoV-2 infects and replicates in cells of the human endocrine and exocrine pancreas (9). Increasing evidence also suggests that SARS-CoV-2 infects endothelial cells and that COVID-19 is a multiorgan endothelial cell disease (14). The evidence of SARS-CoV-2 infection of pancreatic cells, including endothelial cells, and its consequences for pancreatic histopathology remains poorly explored.

In this study, we used publicly available single-cell RNA sequencing (scRNA-seq) data sets, pancreatic sections from male and female controls, SARS-CoV-2–infected nonhuman primates (NHPs), and patients who had died from COVID-19, to assess the expression of ACE2 and TMPRSS2 and localize SARS-CoV-2 infection across pancreatic cells and examine the effect of SARS-CoV-2 infection on pancreatic histopathology.

## Results

### ACE2 protein expression in pancreata of NHPs.

**Figure 1:**
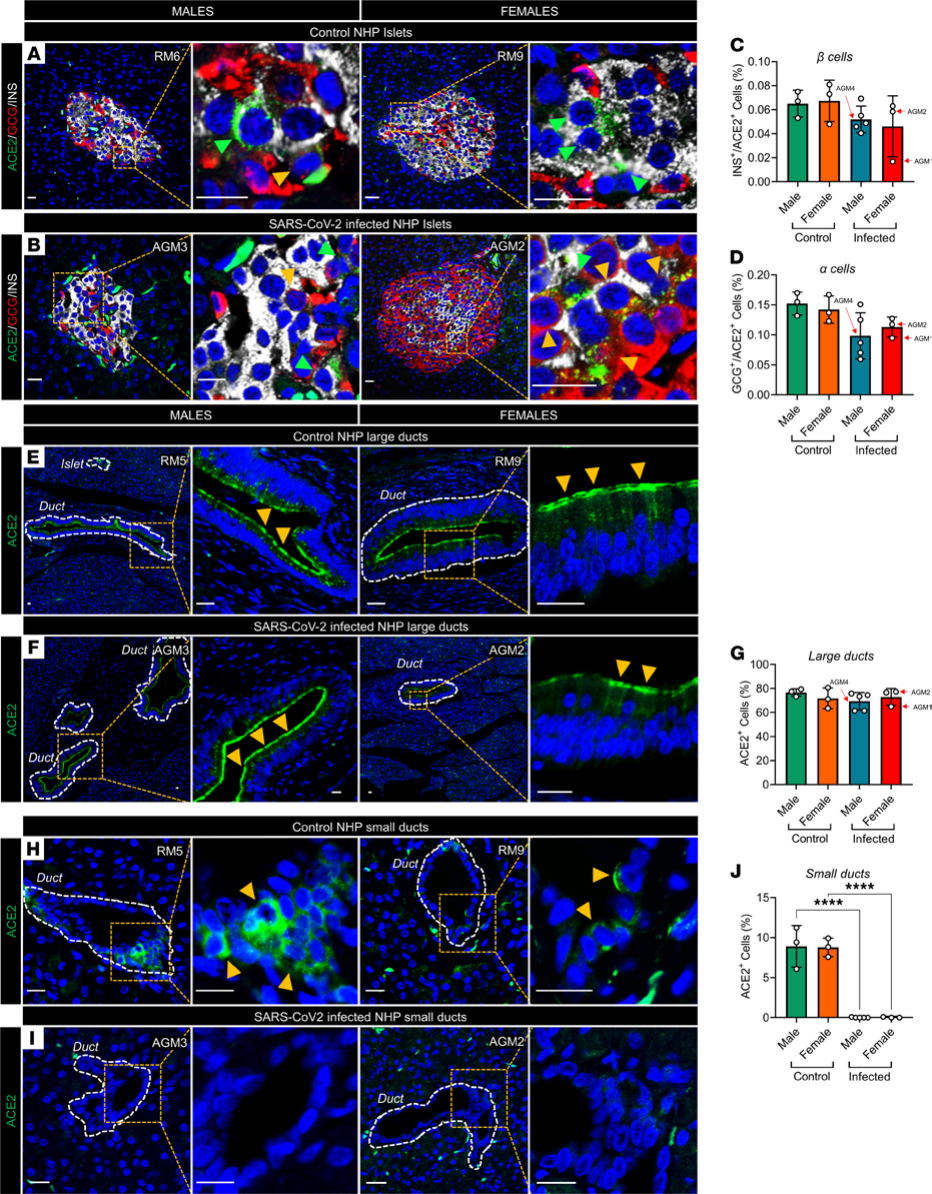
ACE2 protein expression in NHP pancreata. (**A**) Representative confocal immunofluorescence (IF) images of pancreatic islets from uninfected male and female NHPs. Insets show colocalization of insulin (INS) with angiotensin-converting enzyme 2 (ACE2) (green arrows) and glucagon (GCG) with ACE2 (yellow arrows). (**B**) Representative IF images of pancreatic islets from SARS-CoV-2–infected male and female NHPs. Insets show colocalization of INS with ACE2 (green arrows) and GCG with ACE2 (yellow arrows). (**C**) Quantification of β cells expressing ACE2 and (**D**) α cells expressing ACE2 across NHPs. (**E**) Representative IF images of ACE2 expression in large ducts of control NHP and (**F**) SARS-CoV-2–infected pancreas (inset, yellow arrows). (**G**) Quantification of large duct–derived ductal cells expressing ACE2. (**H**) Representative IF images of ACE2 expression in small ducts in control and (**I**) SARS-CoV-2–infected NHP pancreas. (**J**) Quantification of small pancreatic duct–derived ductal cells expressing ACE2. Experiments represent *n =* 3 to 5 biological replicates. Bar graphs show the mean ± SD. Scale bars: 25 μm. ****P < 0.0001 by 1-way ANOVA with post hoc Tukey’s multiple comparison test (**C**, **D**, **G**, and **J**).

We studied ACE2 expression by immunofluorescence using archived pancreatic samples from male and female African green monkeys (AGMs) and rhesus macaques (RMs) infected with SARS-CoV-2 (ref. 15 and Supplemental Figure 1A; supplemental material available online with this article; https://doi.org/10.1172/jci.insight.151551DS1). We observed similar ACE2 immunostaining in rare insulin-expressing (INS^+^) β cells in noninfected controls (Figure 1, A and C) and SARS-CoV-2–infected (Figure 1, B and C) male and female NHPs. ACE2 immunostaining was more pronounced in glucagon-expressing (GCG^+^) α cells from noninfected controls (Figure 1, A and D) and SARS-CoV-2–infected (Figure 1, B and D) male and female NHPs. In the exocrine pancreas, we observed high ACE2 expression in large ducts (large lumen surrounded by stroma) in male and female noninfected controls (Figure 1, E and G) and SARS-CoV-2–infected (Figure 1, F and G) NHPs. Similarly, we detected expression of ACE2 in small ducts (small lumen surrounded by parenchyma) of noninfected control (Figure 1, F and J) male and female NHPs. Notably, ACE2 expression was dramatically decreased in small ducts of male and female SARS-CoV-2–infected NHPs compared with noninfected controls (Figure 1, I and J).

### ACE2 and TMPRSS2 expression in human pancreata.

We studied ACE2 and TMPRSS2 expression across tissues of the Human Protein Atlas (HPA; ref. 16). In bulk-tissue RNA-seq data sets from the HPA, ACE2 mRNA and protein are expressed in both endocrine and ductal cells of the pancreas (Supplemental Figure 1, B and C). This is in agreement with scRNA-seq data sets that also show ACE2 mRNA expression in pancreatic endocrine and ductal cells (Supplemental Figure 1, D–F).

To examine the coexpression of ACE2 and TMPRSS2 in human male compared with female pancreatic cells, we constructed a single-cell expression atlas of the pancreas using 6 scRNA-seq data sets (refs. 17–22 and Supplemental Figure 2, A and B), stratified by sex (Supplemental Figure 2C). We validated sex stratification across cell types (Supplemental Figure 2D) and data sets (Supplemental Figure 2E) based on the classical X and Y chromosome–associated genes XIST and UTY, respectively.

We studied ACE2 and TMPRSS2 coexpression in male and female cells expressing at least 1 count of ACE2 and TMPRSS2 mRNA. These data revealed sex-specific differences in coexpression, including coexpression by specific cell populations in males but not females. Cell types with coexpression of ACE2 and TMPRSS2 observed in both sexes included ductal cells (0.69% in males vs. 0.51% in females), activated stellate cells (0.44% in males vs. 0.05% in females), and acinar cells (0.05% in males vs. 0.12% in females). Cell types of interest showing coexpression of these 2 markers in males, but not females, include quiescent stellate cells (0.17%), macrophages (0.25%), endothelial cells (0.15%), and α cells (0.07%; Supplemental Figure 2, F and G). Notably, using an scRNA-seq data set of chronic pancreatitis (17), we observed the highest ACE2 and TMPRSS2 coexpression in male ductal (8.62%) and β cells (2.06%) (Supplemental Figure 2, H and I).

**Figure 2:**
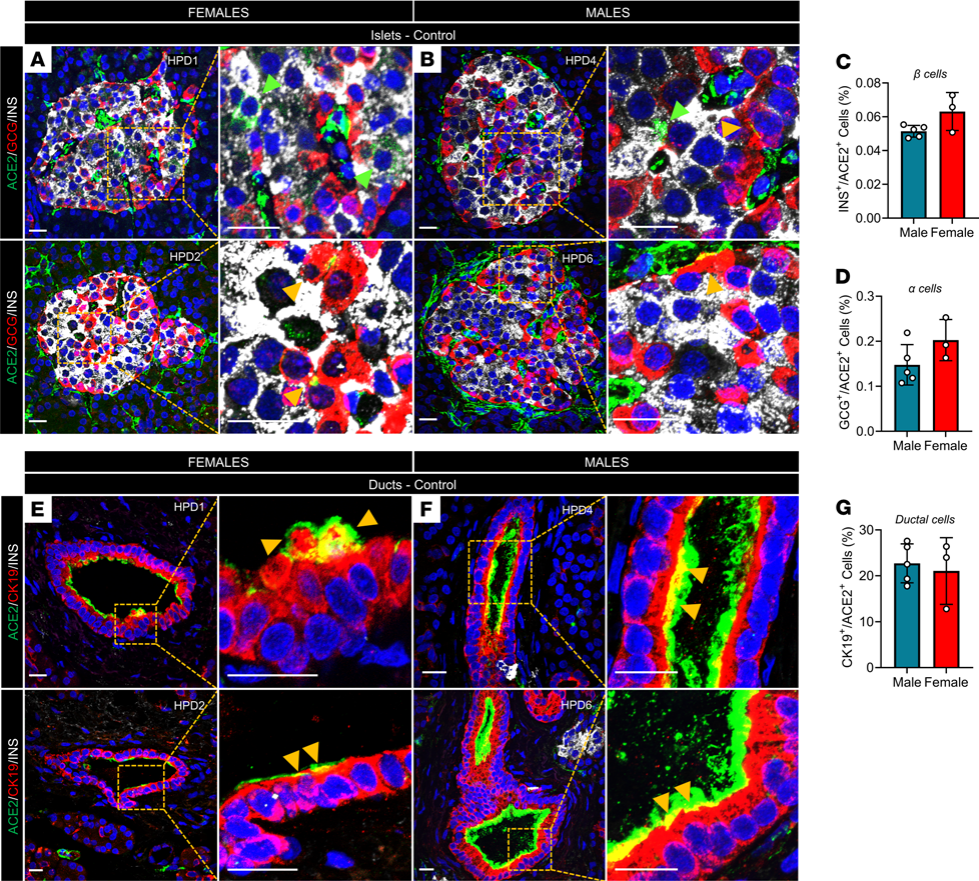
ACE2 protein expression in human pancreata. (**A**) Representative immunofluorescence (IF) images of pancreatic islets from 2 human female and (**B**) male donors, showing ACE2 expression in α and β cells. Insets show colocalization of insulin (INS) with ACE2 (green arrows) and glucagon (GCG) with ACE2 (yellow arrows). (**C**) Quantification of β cells and (**D**) α cells expressing ACE2. (**E**) Representative IF images of pancreatic ducts from 2 human female and (**F**) male donors showing ACE2 expression in ductal cells (CK19). Insets show colocalization of CK19 with ACE2 (yellow arrows). (**G**) Quantification of pancreatic ductal cells expressing ACE2. Experiments represent *n =* 3 biological replicates. Bar graphs show the mean ± SD. Scale bars: 25 μm. Differences were assessed using an unpaired *t* test (**C**, **D**, and **G**).

We studied ACE2 protein expression by immunofluorescence in pancreatic sections from 5 male and 3 female human donors. We observed ACE2 expression in GCG^+^ α cells and to a lesser extent in INS^+^ β cells in both male and female islets, without apparent sex differences (Figure 2, A–D). In the exocrine pancreas, cytokeratin-19–expressing (CK19^+^) ductal cells accounted for the highest expression level of ACE2 across the pancreas in human males and females and without apparent sex differences (Figure 2, E–G).

### SARS-CoV-2 infects pancreatic cells in NHPs.

**Figure 3:**
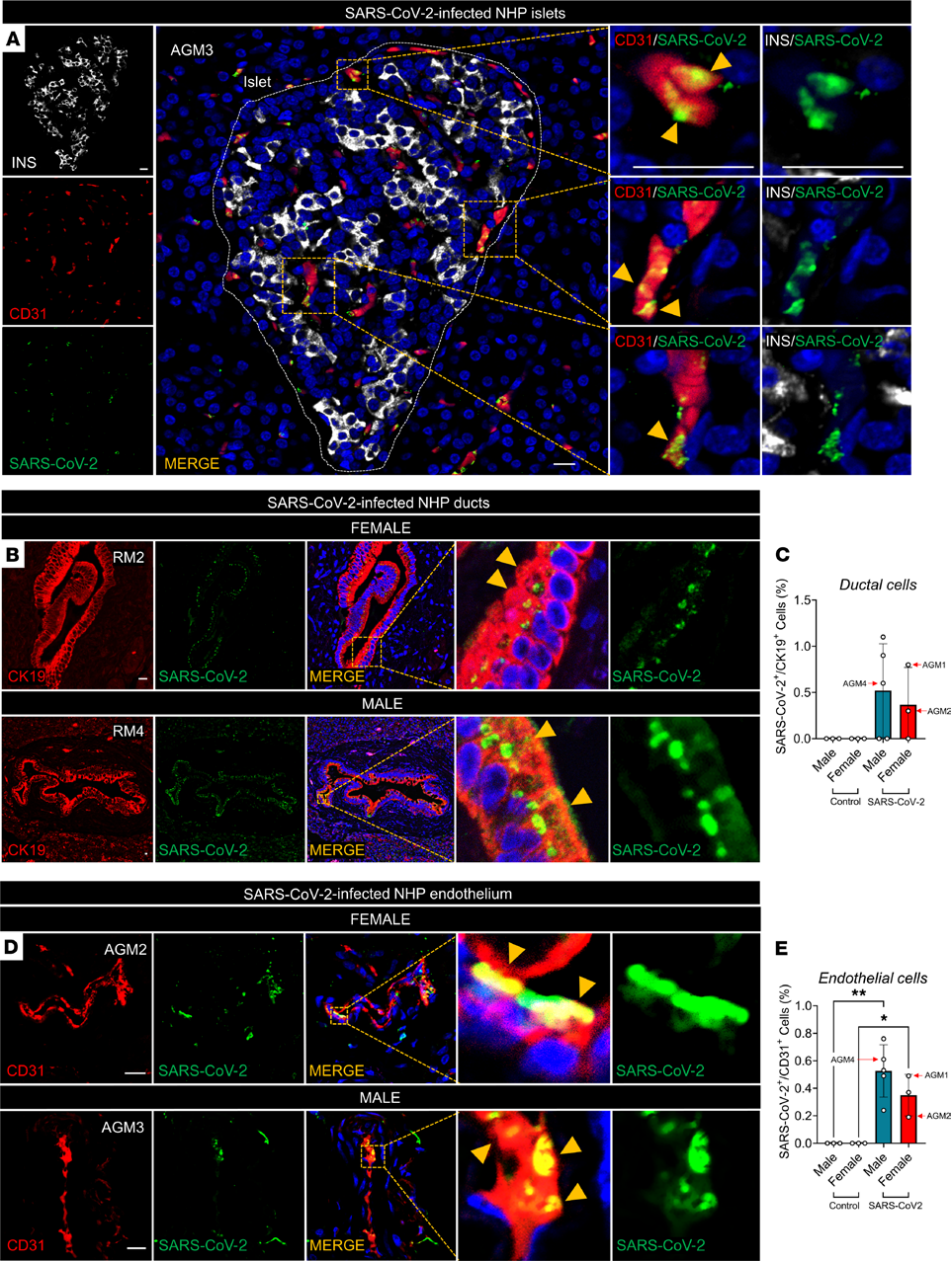
SARS-CoV-2 infects pancreatic ductal and endothelial cells in NHPs. (**A**) Representative immunofluorescence (IF) images of a SARS-CoV-2–infected NHP islet, showing SARS-CoV-2 nucleoprotein (SARS-CoV-2-NP) colocalization with CD31 in endothelial cells. (**B**) Representative IF images of NHP pancreas showing SARS-CoV-2-NP presence in ductal cells. Insets show CK19 and SARS-CoV-2-NP colocalization (yellow arrows). (**C**) Quantification of SARS-CoV-2–infected ductal cells. (**D**) Representative IF image of SARS-CoV-2–infected NHP pancreas showing SARS-CoV-2-NP in CD31^+^ endothelial cells. Insets show colocalization of CK19 with SARS-CoV-2-NP (yellow arrows). (**E**) Quantification of SARS-CoV-2–infected NHP pancreatic endothelium. Experiments represent *n =* 3 to 5 biological replicates. Bar graphs show the mean ± SD. Scale bars: 25 μm. *P < 0.05, **P < 0.01 by 1-way ANOVA with post hoc Tukey’s multiple comparison test (**C** and **E**).

We examined SARS-CoV-2 nucleocapsid protein (SARS-CoV-2-NP) immunopositivity in NHPs’ pancreatic sections. Note that 2 of 8 SARS-CoV-2–infected female NHPs, AGM1 and AGM2, developed severe new-onset diabetes requiring euthanasia 21 days and 9 days after infection, respectively. One infected male exhibited elevated glucose 24 days after infection. Note that the severity of SARS-CoV-2 infection differs significantly in AGMs and RMs (15), which could have precipitated the onset of diabetes in the former but not the latter. None of the noninfected primates developed hyperglycemia (Supplemental Figure 3, A–C). We did not detect any localization of SARS-CoV-2-NP in endocrine α or β cells in 8 male and female SARS-CoV-2–infected NHPs (Figure 3A). However, SARS-CoV-2-NP was coexpressed with platelet endothelial cell adhesion molecule-1 (PECAM1/CD31) in the endothelial cells of the islet microcapillaries in 1 male NHP (Figure 3A). SARS-CoV-2-NP also colocalized with CK19^+^ ductal cells within large ducts across the pancreas to a similar extent in 3 male and the 2 diabetic female SARS-CoV-2–infected NHPs, which we did not observe in noninfected controls (Figure 3, B and C, and Supplemental Figure 3D). Consistent with the disappearance of ACE2 expression in small ducts of SARS-CoV-2–infected NHPs described above (Figure 1, I and J), we observed no immunoreactivity for SARS-CoV-2-NP in small ducts from all SARS-CoV-2–infected NHPs. Notably, among pancreatic cells, we observed the highest SARS-CoV-2-NP immunopositivity in CD31^+^ endothelial cells from small pancreatic arteries in all 8 SARS-CoV-2–infected male and female NPHs, which was not observed in noninfected controls (Figure 3, D and E).

### SARS-CoV-2 infection results in thrombofibrosis of the pancreas in NHPs.

**Figure 4:**
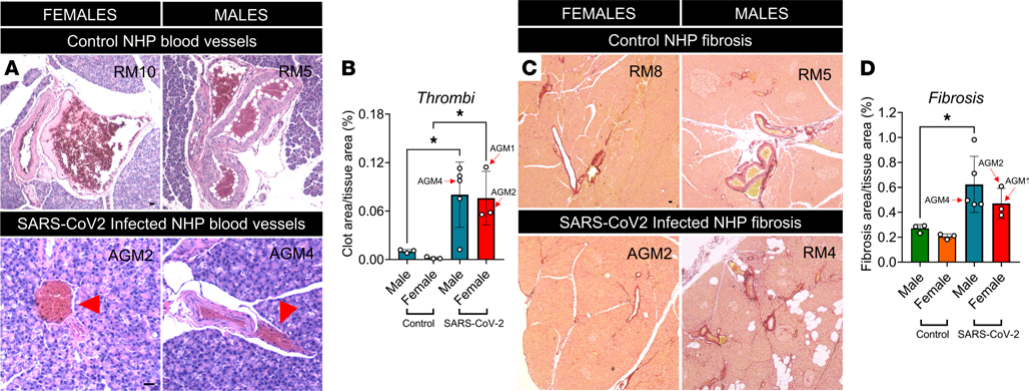
SARS-CoV-2 infection is associated with pancreatic thrombofibrosis in NHPs. (**A**) Representative pancreatic histological sections stained with H&E, showing blood vessels. Images show thrombi in venous compartments of SARS-CoV-2–infected NHP compared with control pancreas. (**B**) Quantification of thrombotic area over pancreatic area, across NHP pancreata. (**C**) Representative pancreatic sections stained with Picrosirius red. (**D**) Quantification showing fibrotic area over pancreatic area. Experiments represent *n =* 3 to 5 biological replicates. Bar graphs show the mean ± SD. Scale bars: 25 μm. *P < 0.05 by 1-way ANOVA with post hoc Tukey’s multiple comparison test (**B** and **D**).

Examination of the entire pancreatic sections stained with hematoxylin and eosin (H&E) revealed multiple microthrombi in small veins across the parenchyma (Figure 4A). The thrombotic areas were higher in SARS-CoV-2–infected male and female NHPs than in noninfected controls (Figure 4B). We next stained pancreatic sections with Picrosirius red (PSR), a classical indicator of collagen and ECM deposition (23), to assess inflammation-induced fibrosis. SARS-CoV-2–infected male NHPs exhibited greater fibrotic areas in comparison with noninfected controls, which did not reach significance in female NHPs (Figure 4, C and D). We also quantified pancreatic lipase, a marker of acute pancreatitis, in serum isolated from infected NHPs. Male and female NHPs exhibited elevated serum lipase in comparison with published normal levels (24) (Supplemental Figure 3E).

### SARS-CoV-2 infects endocrine, exocrine, and endothelial cells in humans.

**Figure 5:**
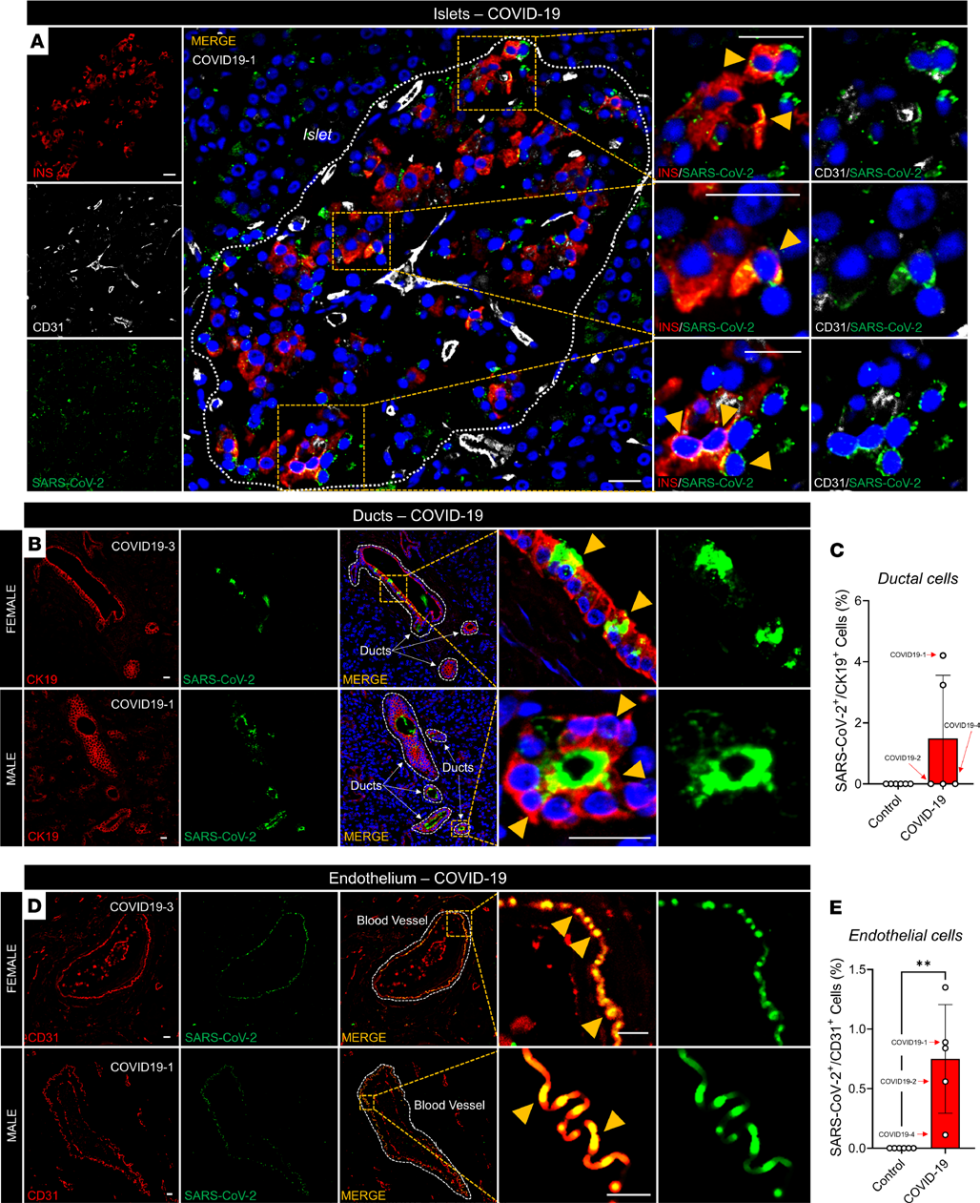
SARS-CoV-2 infects pancreatic endocrine, exocrine, and endothelial cells in humans. (**A**) Representative immunofluorescence (IF) image of a pancreatic islet from a deceased patient with COVID-19 showing SARS-CoV-2-NP in β cells (yellow arrows). (**B**) Representative IF images showing SARS-CoV-2–infected ductal cells (yellow arrows) in deceased COVID-19 subjects. (**C**) Quantification showing the number of SARS-CoV-2–infected ductal cells. (**D**) Representative IF images showing SARS-CoV-2–infected endothelium (yellow arrows). (**E**) Quantification showing the percentage area of infected endothelium, across pancreata. All experiments represent *n =* 3 to 5 biological replicates. Bar graphs show the mean ± SD. Scale bars: 25 μm. **P < 0.01 by unpaired *t* test (**C** and **E**).

**Figure 6:**
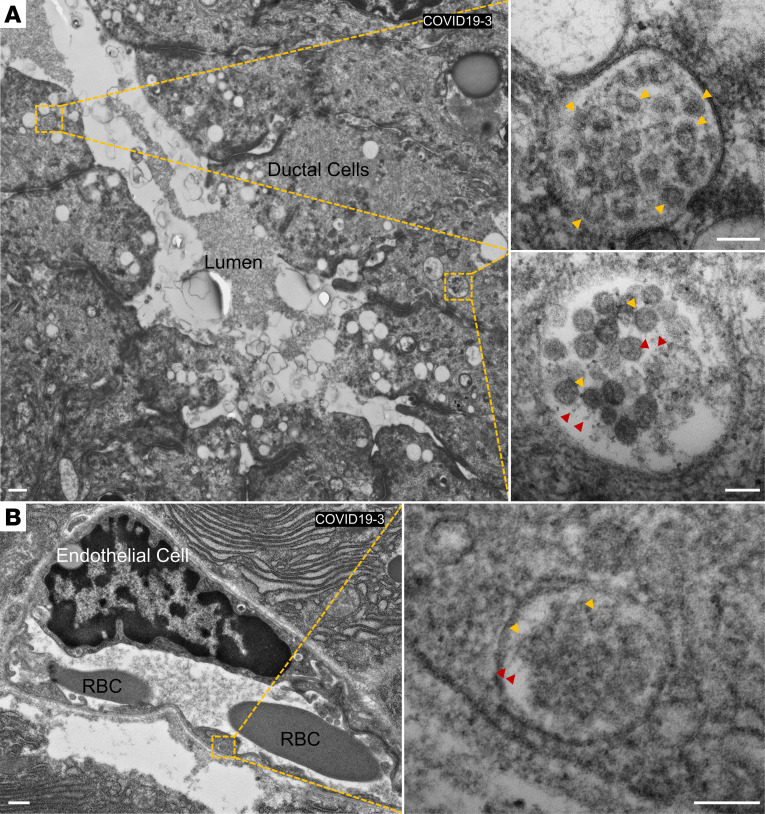
Transmission electron microscopy of SARS-C0V-2 particles in the pancreas of patients with COVID-19. (**A**) Representative transmission electron micrographs of fixed pancreatic tissue. Ductal cells are shown containing SARS-CoV-2 particles (insets). Yellow arrows show SARS-CoV-2 particles and red arrows show the spike protein. (**B**) Representative transmission electron micrographs of endothelial cells in fixed COVID-19 patient pancreas. Endothelial cells show presence of SARS-CoV-2 particles (inset). Yellow arrows show SARS-CoV-2 particles and red arrows show the spike protein. All experiments represent *n =* 3 to 5 biological replicates. Scale bars: 500 nm and 100 nm (insets).

We conducted SARS-CoV-2-NP immunostaining and histological analysis of autopsied pancreatic sections acquired from patients who had died from COVID-19 (Supplemental Table 1). We observed colocalization of SARS-CoV-2-NP with multiple INS^+^ β cells and non–insulin-producing islet cells in 1 male subject (COVID19-1), who was admitted with new-onset diabetes (nonfasting glucose > 300 mg/dL), demonstrating SARS-CoV-2 infection of β cells and other islet cells in this patient (Figure 5A). Similar to our observations in SARS-CoV-2–infected NHPs, we observed colocalization of SARS-CoV-2-NP with CK19^+^ ductal cells in the male subject with new-onset diabetes, and in 1 female subject with a history of type 2 diabetes who had died from COVID-19 (COVID19-3). This was not observed in pancreata from noninfected control subjects, demonstrating that SARS-CoV-2 infected the exocrine pancreatic ducts (Figure 5, B and C). In addition, we observed colocalization for SARS-CoV-2-NP with CD31^+^ endothelial cells in all 5 male and female subjects who had died from COVID-19, including one normoglycemic individual (COVID19-4), which we did not observe in pancreata from noninfected controls (Figure 5, D and E). Additional analysis of pancreatic sections by electron microscopy revealed the presence of SARS-CoV-2 particles in the endothelium and ductal architecture of a female COVID-19 individual (COVID19-3; Figure 6, A and B).

### SARS-CoV-2 infection produces thrombofibrosis and endotheliitis in humans.

**Figure 7:**
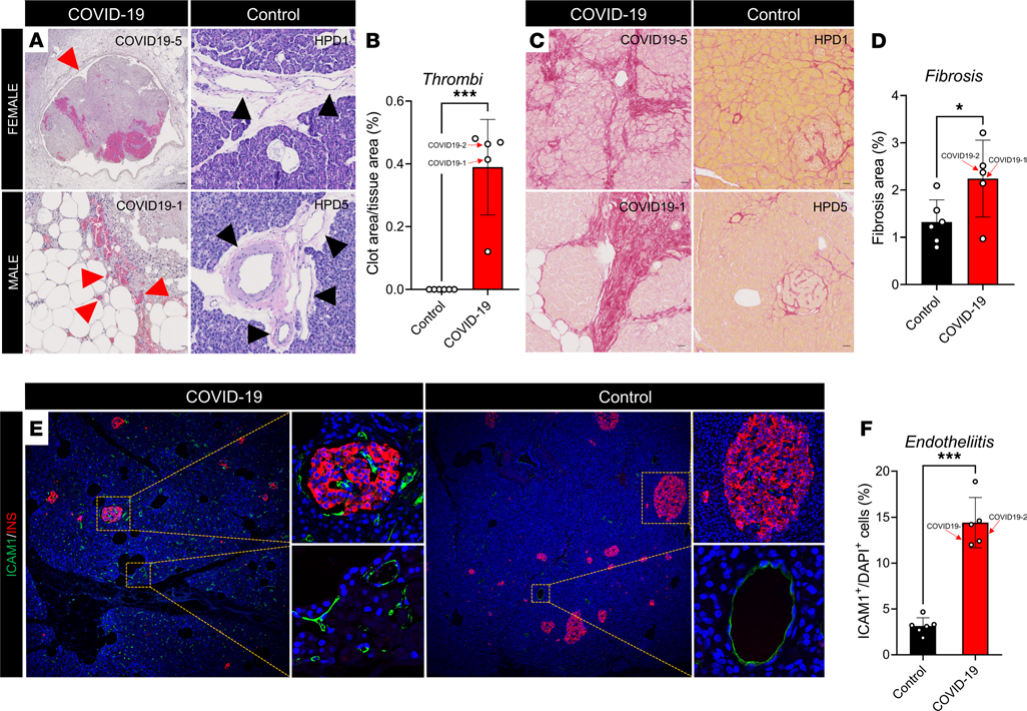
SARS-CoV-2 infection is associated with thrombofibrosis in humans. (**A**) Representative pancreatic sections stained with H&E, showing blood vessels. Images show thrombi in venous compartments in patients with COVID-19 (red arrows) compared with normal pancreatic blood vessels (black arrows). (**B**) Quantification of thrombotic area over total tissue area. (**C**) Representative pancreatic histological sections stained with Picrosirius red. (**D**) Quantification of fibrotic area over total tissue area. (**E**) Representative immunofluorescence images showing the distribution of ICAM1 expression in the endothelium of the exocrine and endocrine pancreas (insets) in patients with COVID-19 (left) and uninfected controls (right). (**F**) Quantification of endotheliitis over total tissue area. All experiments represent *n =* 3 to 5 biological replicates. Bar graphs show the mean ± SD. Scale bars: 25 μm. *P < 0.05, ***P < 0.001 by unpaired *t* test (**B**, **D**, and **F**).

In all male and female subjects who had died from COVID-19, analysis of whole pancreatic sections stained with H&E revealed multiple microthrombi of pancreatic venules (Figure 7, A and B) and PSR staining exposed significantly increased fibrosis (Figure 7, C and D) compared with controls. Pancreatic and islet expression of intercellular adhesion molecule 1 (ICAM1), a marker of endothelial dysfunction and inflammation (25), was increased in individuals with COVID-19 compared with controls, suggesting the presence of endotheliitis of the entire pancreatic vasculature (Figure 7, E and F) and the islet microvasculature (Figure 7G).

## Discussion

Several reports have suggested that COVID-19 is associated with the occurrence of insulin-deficient forms of diabetes, raising the possibility that SARS-CoV-2 produces acute β cell dysfunction (2–4). In this study, 2 out of 8 NHPs developed new-onset hyperglycemia following SARS-CoV-2 infection, and 2 out of 5 patients with COVID-19 exhibited new-onset hyperglycemia at hospital admission. After assembling a pancreatic single-cell expression atlas from 6 publicly available scRNA-seq data sets, we provide information on ACE2 and TMPRSS2 coexpression in human islet cells in males, extending results obtained by others without sex stratification and without coexpression at the same single-cell level (9, 12), and demonstrate that SARS-CoV-2 can directly infect islet cells. Accordingly, two studies reported the presence of SARS-CoV-2 in islets cells, including individual β cells, in male and female subjects who had died from COVID-19 (9, 12). Here, we provide additional evidence that SARS-CoV-2 infected β cells in a male COVID-19 patient who exhibited new-onset diabetes at hospital admission. We also report that SARS-CoV-2 infected the islet microvasculature in a female NHP.

Evidence suggests that COVID-19 produces pancreatic exocrine cell injury, resulting in pancreatitis (6, 26–31). We observed that among pancreatic cells, ductal cells exhibited the highest levels of ACE2 and TMPRSS2 coexpression in the same cells and in both sexes. Accordingly, we showed that SARS-CoV-2 infected ductal cells in male and female NHPs and humans who had died from COVID-19. Further, we observed elevated pancreatic fibrosis in male and female NHPs and humans who had died from COVID-19. During pancreatic inflammation, activated pancreatic stellate cells (PSCs) release inflammatory cytokines and chemokines, inducing ECM deposition, altogether resulting in pancreatic fibrosis (32). Consequently, SARS-CoV-2 infection and inflammation of pancreatic cells may activate PSCs, resulting in fibrosis as observed in the infected NHP and human pancreata.

Evidence also suggests that SARS-CoV-2 infects endothelial cells and that COVID-19 is an endothelial cell disease (14, 33). In severe cases, massive endothelial dysfunction, disseminated coagulopathy, and complement-induced thrombosis produce systemic microangiopathy and thromboembolism (34). Consistent with this concept, we observed expression of ACE2 and TMPRSS2 in pancreatic endothelial cells. Surprisingly, as in the case of islet cells, we observed coexpression of ACE2 and TMPRSS2 in the same cells in males only. The absence of ACE2 and TMPRSS2 coexpression in the same female islet and endothelial cells from the scRNA-seq data sets should be interpreted with caution, as it could be an artifact derived from the lack of sufficient sequencing depth of the scRNA-seq. Nevertheless, we observed SARS-CoV-2 infection of islet microvasculature in female SARS-CoV-2–infected NHPs. We did not observe SARS-CoV-2 in human islet capillaries, but SARS-CoV-2 infected the microvasculature of the exocrine pancreas in all male and female NHPs and humans who had died from COVID-19. Notably, in NHPs and humans, SARS-CoV-2 infection was associated with multiple thrombi of microvessels and with markers of endotheliitis in humans. To what extent endotheliitis and thrombosis are a direct effect of viral endothelial cell infection and dysfunction, or are the consequence of immune-induced thrombosis, requires further studies. Together, the infection of ductal and endothelial cells could produce indirect β cell insult and dysfunction via proximal inflammation. Indeed, 1 female patient with COVID-19 exhibited new-onset diabetes at admission, and 2 female NHPs developed new-onset diabetes days after SARS-CoV-2 infection, without SARS-CoV-2 infection of islets.

In summary, SARS-CoV-2 infects pancreatic islet, ductal ,and endothelial cells in male and female NHPs and humans with COVID-19. In both species, SARS-CoV-2 infection is associated with disseminated pancreatic endotheliitis, microthrombi, fibrosis, and new-onset hyperglycemia, suggesting that COVID-19 produces new-onset diabetes. Most importantly, the long-term consequences of a fibrotic/thrombotic pancreas, such as chronic pancreatic exocrine dysfunction and late-onset diabetes, should be investigated as postacute sequelae of COVID-19.

## Methods

More information is available in the supplemental methods.

### Immunofluorescence staining.

Tissue sections were stained as previously described in Qadir et al. (35).

### Study approval.

All studies were approved by the Institutional Animal Care and Use Committee and Institutional Biosafety Committee of Tulane University. The Tulane National Primate Research Center is fully accredited by the American Association for Accreditation of Laboratory Animal Care.

## Author contributions

MMFQ and FMJ designed the study. MMFQ, MB, IB, and DDG performed experiments, analyzed data, and performed statistical analyses. WB performed tissue processing, imaging and analysis for electron microscopy. LADM, TF, RVB, RB, and JR developed the NHP models and provided NHP archived pancreatic sections. MMFQ and FMJ wrote the manuscript. RSVH, TF, LADM, JR, EL, JKK, and XQ reviewed the manuscript. All authors approved the final manuscript. FMJ accepts responsibility for the overall content of this work and ensures all statements in the manuscript are true to his knowledge.

## Supplementary Material

Supplemental data

## Version 1 07/09/2021

In-Press Preview

## Version 2 08/23/2021

Electronic publication

## Version 3 08/26/2021

Graphical abstract added.
