# Supplementary material for: SARS-CoV-2 infection of the pancreas promotes thrombofibrosis and is associated with new-onset diabetes
Source: JCI Insight. 2021 Aug 23;6(16):e151551. doi: 10.1172/jci.insight.151551 (PMC8410013; doi:10.1172/jci.insight.151551)
Supplement: Supplemental data [file jciinsight-6-151551-s275.pdf]

Supplemental material

SARS-CoV-2 infection of the pancreas promotes thrombo-fibrosis and is associated with new-onset diabetes.

Table of contents:

Supplemental Figure 1

Supplemental Figure 2

Supplemental Figure 3

Supplemental Table 1

Supplemental methods

Supplemental Table 2

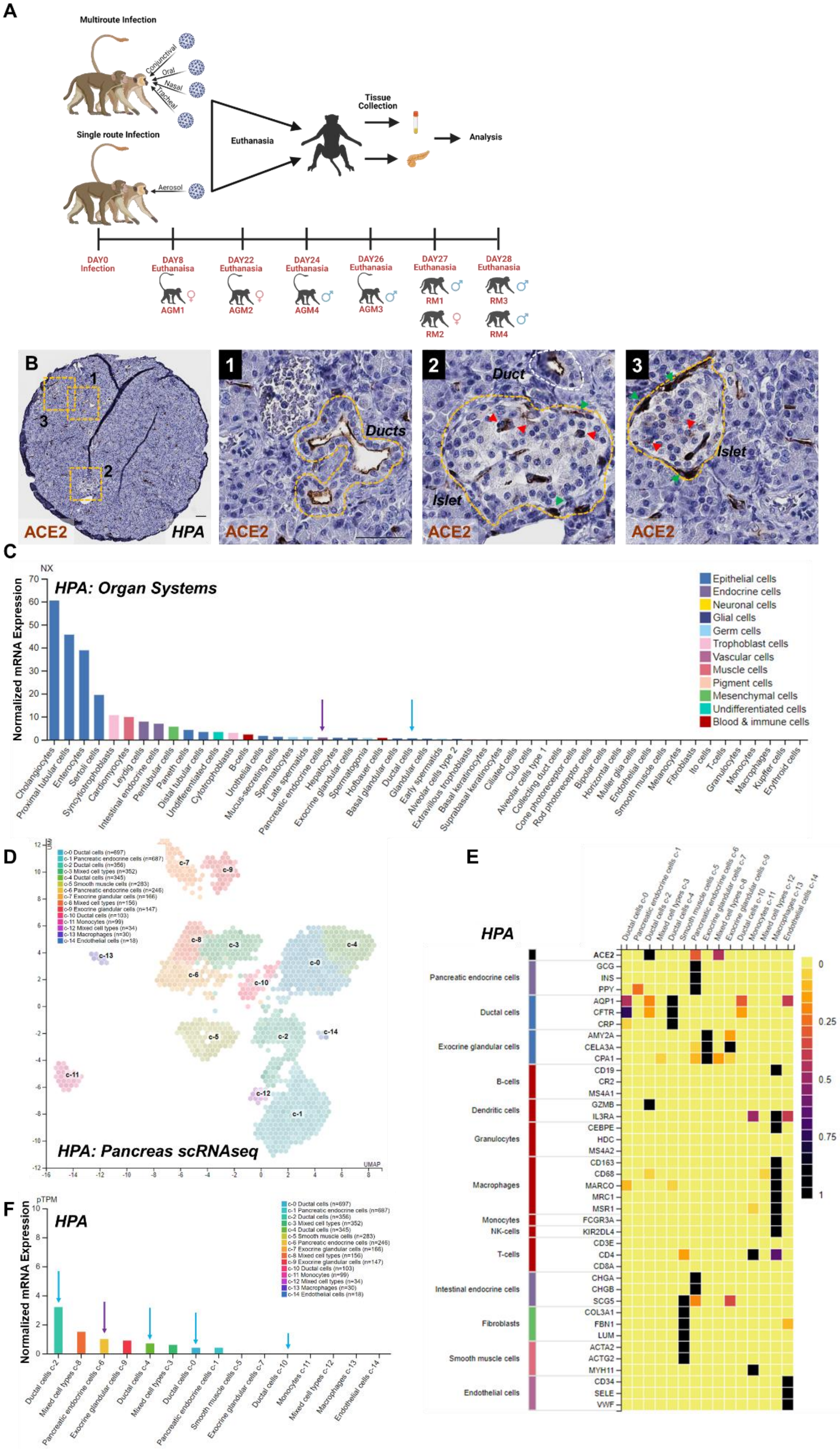
A
Multiroute Infection
Conjunctival
Oral
Nasal
Tracheal
Single route Infection
Aerosol
Euthanasia
Tissue Collection
Analysis
DAY0 Infection
DAY8 Euthanaisa
DAY22 Euthanasia
DAY24 Euthanasia
DAY26 Euthanasia
DAY27 Euthanasia
DAY28 Euthanasia
AGM1
AGM2
AGM4
AGM3
RM1
RM2
RM3
RM4
B
ACE2
HPA
Ducts
Duct
Islet
C
HPA: Organ Systems
Normalized mRNA Expression
NX
Epithelial cells
Endocrine cells
Neuronal cells
Glial cells
Germ cells
Trophoblast cells
Vascular cells
Muscle cells
Pigment cells
Mesenchymal cells
Undifferentiated cells
Blood & immune cells
D
HPA: Pancreas scRNAseq
UMAP
E
HPA
F
HPA
pTPM

Supplemental Figure 1. Study design for NHP experiments and ACE2 expression signature across human cell types based on Human Protein Atlas and the Genotype-Tissue Expression portal. (A) Study design showing the administration of virus to male and female African green monkeys (AGM) and Rhesus macaque (RM) NHPs. At the designated euthanasia points, peripheral blood and pancreatic tissue was recovered and fixed for immunostaining and histological analysis. **(B)** Representative H/E histological imaging of a pancreatic tissue section stained for ACE2. Insets showing ACE2 in ducts (1), Islet resident endocrine cells (2, 3: red arrows) and stellate cells (2, 3: green arrows). **(C)** scRNAseq expression plots showing normalized gene expression (NX) for ACE2 across all major cell types of the human body including pancreatic endocrine cells (purple arrow) and ductal cells (blue arrow). **(D)** Uniform Manifold approximation and projection (UMAP) plot showing scRNAseq of HPA derived pancreas data. **(E)** Heatmap showing relative mRNA expression across cells in (D). Data shows UMAP defined cell clusters (x-axis) and known canonical cell type specific markers (y-axis). **(F)** Normalized gene expression of ACE2 mRNA across clusters shown in (D). High expression levels can be seen in ductal cell types (blue arrows) and endocrine cells (purple arrows). Scale bars: 50µm. Images in B downloaded from: https://www.proteinatlas.org/ENSG00000130234-ACE2/tissue/pancreas#img, image in C downloaded from: https://www.proteinatlas.org/ENSG00000130234-ACE2/celltype, image in D-F downloaded from https://www.proteinatlas.org/ENSG00000130234-ACE2/celltype/pancreas, on 3/15/2021. Image credits (B-E): Human Protein Atlas v20.1 https://www.proteinatlas.org/. (A) is created using biorender.com.

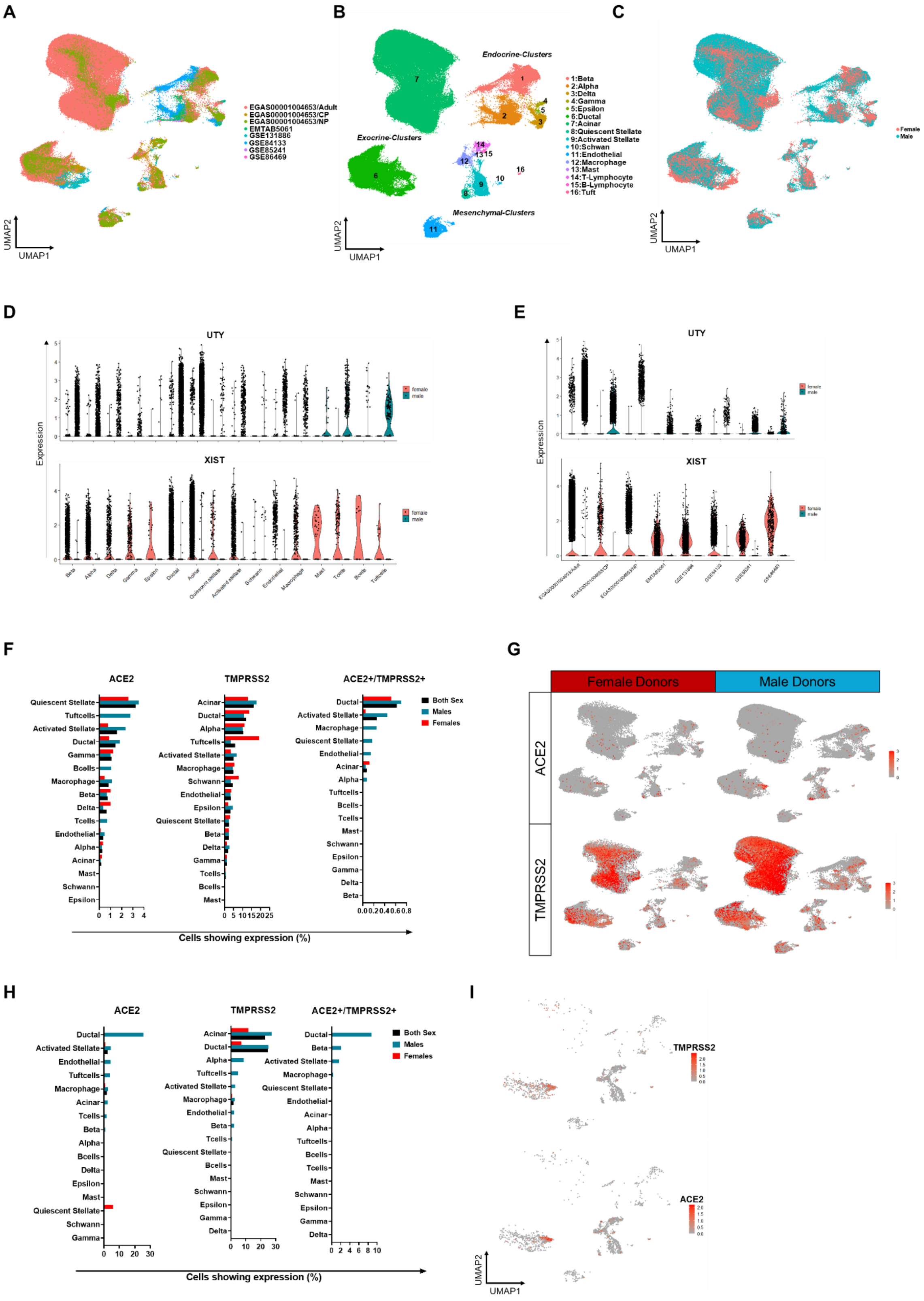
A
EGAS00001004653/Adult
EGAS00001004653/CP
EGAS00001004653/NP
EMTAB5061
GSE131886
GSE84133
GSE85241
GSE86469
UMAP2
UMAP1
B
Endocrine-Clusters
Exocrine-Clusters
Mesenchymal-Clusters
1:Beta
2:Alpha
3:Delta
4:Gamma
5:Epsilon
6:Ductal
7:Acinar
8:Quiescent Stellate
9:Activated Stellate
10:Schwan
11:Endothelial
12:Macrophage
13:Mast
14:T-Lymphocyte
15:B-Lymphocyte
16:Tuft
C
Female
Male
D
UTY
XIST
Expression
female
male
Beta
Alpha
Delta
Gamma
Epsilon
Ductal
Acinar
Quiescent stellate
Activated stellate
Schwann
Endothelial
Macrophage
Mast
Tcells
Bcells
Tuftcells
E
UTY
XIST
Expression
F
ACE2
TMPRSS2
ACE2+/TMPRSS2+
Both Sex
Males
Females
Cells showing expression (%)
G
Female Donors
Male Donors
ACE2
TMPRSS2
H
ACE2
TMPRSS2
ACE2+/TMPRSS2+
Both Sex
Males
Females
Cells showing expression (%)
I
TMPRSS2
ACE2
UMAP2
UMAP1

Supplemental Figure 2. ACE2 and TMPRSS2 RNA co-expression across male and female human pancreas. (A) An integrated uniform multidimensional projection (UMAP) plot 143,362 single cells across 6 publicly available single cell RNA sequencing datasets colored based on the study. Each dot represents the transcriptome of a single cell. The EGAS00001004653 study is further stratified into single nuclei stemming from adults, chronic pancreatitis donors (CP) and neonatal pancreas samples (NP). (n = 16 male + 11 female donors) **(B)** A UMAP plot of data shown in A further classified based on pancreatic cell types. Cell clusters can be stratified into Endocrine-clusters (1-5), ductal clusters (6-7), and mesenchymal clusters (8-16). **(C)** A UMAP plot of data shown in (A and B) further classified based on sex. **(D)** Violin plots showing mRNA expression of male specific Y-chromosome linked UTY and, female specific X-chromosome linked XIST mRNA, across clusters for both sexes. **(E)** Violin plots showing mRNA expression of male specific Y-chromosome linked UTY and, female specific X-chromosome linked XIST mRNA, across datasets for both sexes. **(F)** Gene expression correlation analysis of all cell types, showing the percentage of cells (left to right) expressing ACE2, TMPRSS2 and ACE2/TMPRSS2 co-expression across all pancreatic cell types. Expression is shown across both males and females. In instances where only one sex shows expression a value for both sexes is not calculated or shown. **(G)** UMAP plots showing single cell gene expression of ACE2 and TMPRSS2 across all cells in males and females. **(H)** Gene expression correlation analysis of all cell types, showing the percentage of cells (left to right) expressing ACE2, TMPRSS2 and ACE2/TMPRSS2 co-expression across all pancreatic cell types, within the EGAS00001004653 - chronic pancreatitis dataset. Expression is shown across both males and females. In instances where only one sex shows expression a value for both sexes is not calculated or shown. **(I)** UMAP plots showing expression of TMPRSS2 and ACE2 across the EGAS00001004653 - chronic pancreatitis dataset.

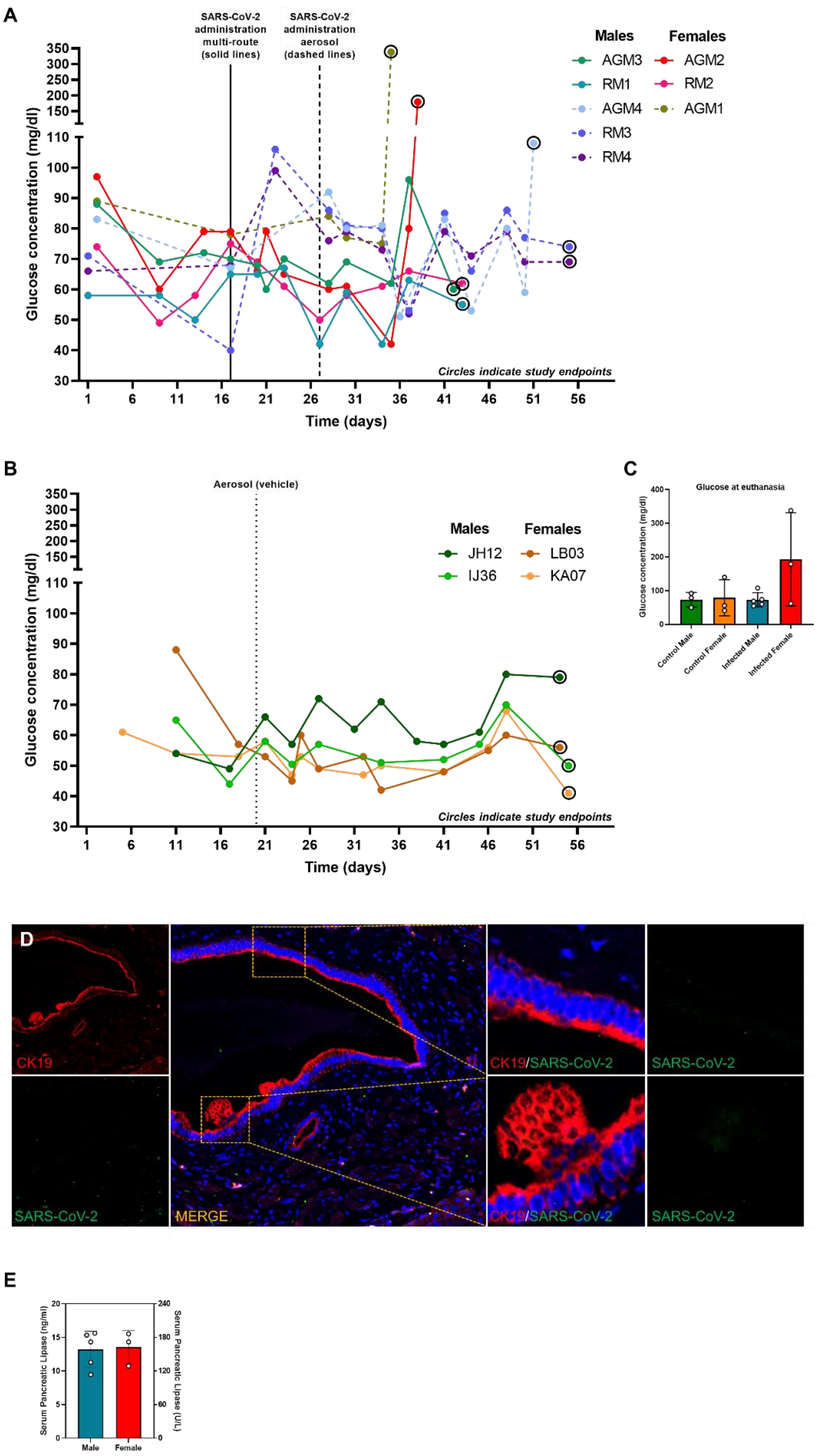
A
SARS-CoV-2 administration multi-route (solid lines)
SARS-CoV-2 administration aerosol (dashed lines)
Males
Females
AGM3
RM1
AGM4
RM3
RM4
AGM2
RM2
AGM1
Glucose concentration (mg/dl)
Circles indicate study endpoints
Time (days)
B
Aerosol (vehicle)
Males
Females
JH12
IJ36
LB03
KA07
Glucose concentration (mg/dl)
Circles indicate study endpoints
Time (days)
C
Glucose at euthanasia
Glucose concentration (mg/dl)
Control Male
Control Female
Infected Male
Infected Female
D
CK19
SARS-CoV-2
MERGE
CK19/SARS-CoV-2
E
Serum Pancreatic Lipase (ng/ml)
Serum Pancreatic Lipase (U/L)
Male
Female

Supplemental Figure 3. Control NHPs do not show containing for SARS-CoV-2 virus. (A) Blood glucose levels of SARS-CoV-2 infected NHPs over time. NHPs treated via multi-route (solid lines) and aerosol (dashed lines) are shown. **(B)** Blood glucose levels of study controls. **(C)** Terminal blood glucose readings of SARS-CoV-2 infected and uninfected NHPs. **(D)** representative confocal maximum projection of a NHP pancreatic duct stained for CK19, showing no colocalization of SARS-CoV-2 Nucleoprotein. **(E)** Bar plot showing quantification of serum pancreatic lipase levels in SARS-CoV-2 infected NHPs. n=3-5 biological replicates. Scale bar: 50μm.

Supplemental Table 1. Donor characteristics table. Table showing the demographics of all the individual donors used in this study. Abbreviations: Years (yrs), Human Pancreatic donor (HPD), Body Mass Index (BMI), Hemoglobin A1c (HbA1c), non-fasting glucose (NFG), reverse transcriptase polymerase chain reaction (RT-PCR), Louisiana State University Health Sciences Center (LSUHSC).

Donor ID	Sex	Age (yrs)	Race/Ethnicity	BMI (Kg/m^2)	HbA1c (%)	Diabetes History	SARS-CoV-2 Confirmation Test	Source
**HPD1**	**Female**	**60**	**Non-Hispanic white**	**25.8**	**5.1**	**No diabetes history**	**Negative(-) SARS-CoV-2 RT-PCR**	**Prodo-labs LA.**
**HPD2**	**Female**	**41**	**Non-Hispanic white**	**30.3**	**4.8**	**No diabetes history**	**Negative(-) SARS-CoV-2 RT-PCR**	**Prodo-labs LA.**
**HPD3**	**Female**	**42**	**Non-Hispanic white**	**23.5**	**5.4**	**No diabetes history**	**Negative(-) SARS-CoV-2 RT-PCR**	**Prodo-labs LA.**
**HPD4**	**Male**	**22**	**Hispanic**	**31.9**	**5.4**	**No diabetes history**	**Negative(-) SARS-CoV-2 RT-PCR**	**Prodo-labs LA.**
**HPD5**	**Male**	**42**	**Non-Hispanic white**	**28.1**	**4.9**	**No diabetes history**	**Negative(-) SARS-CoV-2 RT-PCR**	**Prodo-labs LA.**
**HPD6**	**Male**	**48**	**Non-Hispanic black**	**20.2**	**5.2**	**No diabetes history**	**Negative(-) SARS-CoV-2 RT-PCR**	**Prodo-labs LA.**
**COVID19-1**	**Male**	**61**	**Hispanic**	**22.6**	**N/A**	**No diabetes history NFG>300mg/dL (at admission)**	**Positive(+) SARS-CoV-2 RT-PCR**	**LSUHSC**
**COVID19-2**	**Female**	**72**	**Hispanic**	**40.5**	**N/A**	**No diabetes history NFG>300mg/dL (at admission)**	**Positive(+) SARS-CoV-2 RT-PCR**	**LSUHSC**
**COVID19-3**	**Female**	**73**	**Non-Hispanic white**	**26.2**	**N/A**	**Type-II Diabetes**	**Positive(+) SARS-CoV-2 RT-PCR**	**LSUHSC**
**COVID19-4**	**Female**	**77**	**Non-Hispanic black**	**30.2**	**N/A**	**No diabetes history**	**Positive(+) SARS-CoV-2 RT-PCR**	**LSUHSC**
**COVID19-5**	**Female**	**49**	**Non-Hispanic black**	**48.0**	**N/A**	**Type-II Diabetes**	**Positive(+) SARS-CoV-2 RT-PCR**	**LSUHSC**

Supplemental methods

Materials availability

This study did not generate new reagents. Tissues used in this study were obtained from Prodo Labs Inc, (City of Hope, LA) and from autopsies of deceased individuals with COVID-19. Donor demographics are provided (Supplemental Table 1).

Study approval

The Institutional Animal Care and Use Committee of Tulane University extensively reviewed and subsequently approved all the procedures for these studies. The Tulane National Primate Research Center (TNPRC) is fully accredited by the American Association for Accreditation of Laboratory Animal Care. All animals were cared for in accordance with NIH's Guide for the Care and Use of Laboratory Animals (1). Animal studies were performed within animal biosafety level 3 (ABSL3) containment in the Regional Biocontainment Laboratory at the TNPRC. The Tulane University Institutional Biosafety Committee approved the procedures for sample handling, inactivation, and removal from BSL3 containment.

Control human pancreatic tissue

Tissue biopsies from transplant quality human pancreatic tissue was procured from Prodo Labs. Inc. (City of Hope) from 6 human individuals, free from diabetes and testing negative for SARS-CoV-2 virus via reverse transcriptase polymerase chain reaction at the time of death (Supplemental Table 1). Use of these tissues for research is approved by the Institutional Review Board at Tulane University, the United Network for Organ Sharing (UNOS) and according to federal guidelines with informed consent obtained from each donor's legal representative. Tissue biopsies were fixed in 4% formalin and transported to Tulane University within 24 hours of fixation.

Autopsy subjects and sample processing

Pancreas tissue was preserved from ten individuals who tested positive for SARS-CoV-2 by reverse transcription polymerase chain reaction (RT-PCR) test within 24-48 hrs of death at the University Medical Center New Orleans (New Orleans, LA). Consent for autopsy of persons with COVID-19 was provided by next of kin, and studies within this report are deemed exempt from oversight by the institutional review board at Louisiana State University Health Sciences Center. Upon histopathological evaluation biopsy samples from 5 individuals were excluded owing to extensive autolysis rendering the biopsy tissue of these individuals inappropriate for histological and immunological analysis.

Non-human primates

A total of eight animals, 5 males and 3 females, consisting of 4 AGMs and 4 RMs were used in this study. Animals were exposed to virus via multi-route or aerosol, both modalities resulted in ARDS, as described previously (2). When humane or study endpoints were reached, the animals were euthanized and their pancreas recovered and divided into the tail, body and head sections and stored in fixative (Z-fix, containing 3.7-4% formaldehyde) for a minimum of 72 hours prior to histological evaluation.

Viruses

All animals were challenged with the first US isolate of SARS-CoV-2, 2019-nCoV/USA-WA1/2020 (*https://www.ncbi.nlm.nih.gov/nuccore*; accession number MN985325.1) (3). Virus was prepared in Vero E6 cells and sequenced for validity. Infectivity was evaluated via plaque forming assays in Vero E6 cells, acquired from ATCC (Cat# CRL-1586).

Single-cell RNA sequencing analysis

Single cell RNAseq analysis was performed using Seurat v3.2.2.9002. Six publicly available single cell RNAseq datasets of human pancreatic islet cells (4-7), ductal cells (8), and entire pancreas cells (9) were organized with metadata (study of origin, sex, disease specific stratification) and converted and compiled into a list of Seurat objects. This atlas is composed of 143,362 single cells and spans 16 male and 11 female donors. Following recommended guidelines on sample integration using reciprocal PCA (rPCA) for large datasets (*https://satijalab.org/seurat/archive/v3.2/integration.html*) we integrated and classified cell clusters based on transcriptional similarity to known transcriptional identities of pancreatic cell types.

Data and code availability

Complete computational pipeline for scRNAseq analysis is available for download as R-scripts at the FMJ lab Github as a R code repository: https://github.com/fmjlab/Pancreas_atlas_COVID19. Mapped and optimized Seurat objects (.rds file) of the human pancreas atlas are available upon request.

Immunofluorescence, H&E and PSR staining and image acquisition

Formalin fixed paraffin embedded tissues (FFPE) were sectioned, loaded onto charged slides, de-paraffinized, permeabilized, blocked, and stained as previously described (8, 10). Images were acquired using a Nikon A1R HD25 confocal microscope, using a pinhole setting of 1.2AU and laser intensity set at 2%. H&E and PSR staining was performed on FFPE tissues according to standard methodology at the histology core lab at Tulane University. Brightfield images were acquired using a Axiovision Z1 slide scanner at default 40x brightfield settings recommended by the manufacturer. Images were acquired and processed using Zeiss Zen 3.3 blue software.

Image Processing and Quantification

For analysis involving structures (ducts, islets, blood vessels) at least 20 structures for each donor were randomly selected and quantified. For all analyses in this study images were acquired at either 10x or 20x and at least 10 random sections from each slide, of each donor were selected for downstream analysis. Images were processed using Fiji image J, colocalization were calculated using Fiji image J plugins Coloc2 (11) and EzColocalization (12) plugins. Colocalization was performed by opening .Tiff files of each channel in Fiji image J and two selected fluorescent channels were selected in 'Inputs' tab as the 'reporter.1 (Ch.1)' and 'reporter.2 (Ch.2)' input specifier respectively. DAPI was selected as the 'cell identification input' and reports were aligned and verified by 'Show Threshold' and 'preview' options. We select 'Area' in the filters option as 50-800 pixels to encompass the area of a fluorescent cell, we also select the watershed option to segment areas where 2 or more cells lie near one another. We continuously use 'preview' to verify the software was accurately selecting cells, after each step. Finally, in the 'analysis' tab we select the threshold overlap score (TOS) and the fluorescence threshold (FT) options so that only the top 10% of all pixel intensities are selected for both channels (the top 10% is recommended and in our experience accurately differentiates actual fluorescence vs. artifactual background). After this we further select the 'Average signal', 'Summary', 'Histogram', 'Masks' and 'ROIs' options and use the 'Analyze' button to output a matrix of summary metrics which contains a linear TOS value. We then copy this table in excel and calculate the total number of cells which have a linear TOS value greater than 0.25. This provides the number for cells having a co-localization. We then perform the above for only one protein channel to find the total number of cells expressing a protein (for example only INS and DAPI channels to fine Insulin expressing cells). After evaluating these sets of cells we use the formula (Protein1 and Protein2 colocalizing cells)/(Protein2 cells) to calculate the number of cells expressing a particular protein1 as a function of all cells expressing Protein2. We validated these data by selecting cells detected on random expressing a particular protein and using Fiji ImageJ's ROI function to select cells and use the Coloc2 plugin,

to select each channel and view the results of the ‘Manders’ Correlation’, ‘Spearman Rank Correlation’, ‘2D intensity Histogram’ and ‘Costes’ Significance Test’ which generates a scatterplot and colocalization metrics. If cells had a Costes Probability value (P-Value) of >0.8 then cells were termed to have correctly been identified for colocalization by the plugin EzColocalization. It is advised to use computationally tenacious hardware to enable optimal image quantification analysis. Our system configuration was the following: Intel core i9-9900 CPU, 64 GB DDR3 RAM, Nvidia Quadro P1000 GPU, Windows 10 Enterprise 64 bit. Fiji Image J was manually configured to utilize 16 threads and 60 GB (decimal units). Total clot area was calculated manually using the freehand ROI tool in Fiji image J and then area was calculated using the measure feature. Total PSR regions were calculated using custom designed macros as described previously (13).

Transmission Electron Microscopy

For ultrastructural analyses, tissue sections were deparaffinized in three changes of xylene followed by rehydration in a graded series of ethanol. The tissue samples were then refixed in 2% paraformaldehyde/2.5% glutaraldehyde (Ted Pella Inc.) in 100 mM sodium cacodylate buffer, pH 7.2 for 2 hr at room temperature. Samples were washed in sodium cacodylate buffer and postfixed in 1% osmium tetroxide (Polysciences Inc.) for 1 hr. Samples were then rinsed extensively in dH20 prior to en bloc staining with 1% aqueous uranyl acetate (Ted Pella Inc., Redding, CA) for 1 hr. Following several rinses in dH20, samples were dehydrated in a graded series of ethanol and embedded in Eponate 12 resin (Ted Pella Inc.). Sections of 95 nm were cut with a Leica Ultracut UCT ultramicrotome (Leica Microsystems Inc., Bannockburn, IL), stained with uranyl acetate and lead citrate, and viewed on a JEOL 1200 EX transmission electron microscope (JEOL USA Inc., Peabody, MA) equipped with an AMT 8 megapixel digital camera and AMT Image Capture Engine V602 software (Advanced Microscopy Techniques, Woburn, MA).

Pancreatic Lipase measurements using ELISA

Serum samples from infected NHPs were thawed on ice and subjected to ELISAs for quantitative measurements of serum pancreatic amylase and lipase based on the manufacturer's recommendations. Samples were then corelated to a standard curve and quantified.

Data plotting and graphing

For plotting data into box plots, GraphPad Prism v9 and v8 was utilized. All other graphing and analysis were performed using custom designed scripts in R and the integrated graphics functions [ggplot2] and [RShiny].

Statistical analysis

Following the Shapiro-Wilk normality test, statistical differences between groups were tested using a one-way ANOVA with a Tukey's post-hoc test across multiple groups, or for single comparisons between two groups an unpaired two-tailed t-test was used, with confidence intervals for both tests taken to be 95% (α=0.05).

Supplemental Table 2: Reagents and equipment.

Reagent/Resource	Source	ID
**Antibodies**		
FLEX Polyclonal Guinea Pig Anti-Insulin, Ready to-Use antibody (1:10)	Dako/ Agilent	Cat# IR00261-2
Anti-Glucagon-Mouse Polyclonal (1:100)	R&D Systems	Cat# MAB1249
Anti-KRT19-Mouse Polyclonal (1:50)	Dako/ Agilent	Cat# M0888
Anti-KRT19-Rabbit Polyclonal (1:50)	Abcam	Cat# ab15463-1
Anti-CD31-Mouse Monoclonal (1:100)	BD Biosciences	Cat# 550389
Anti-CD31-Mouse Monoclonal (1:50)	Novus Biologicals	Cat# NBP2-44342
Anti-ACE2-Goat Polyclonal (1:50)	R&D Systems	Cat# AF933
Anti-SARS-CoV2-Nucleocapsid Protein-Rabbit Polyclonal (1:100)	Novus Biologicals	Cat# NB100-56576
Anti-ICAM1-Sheep Polyclonal (1:100)	R&D Systems	Cat# AF720
Anti-CD45-Sheep Polyclonal (1:50)	R&D Systems	Cat# MAB1430
Alexa Fluor® 594 AffiniPure Donkey AntiGuinea Pig IgG (H+L) (1:400)	Jackson Immuno Research laboratories, Inc.	Cat# 706-585-148
Alexa Fluor® 647 AffiniPure Donkey AntiGuinea Pig IgG (H+L) (1:400)	Jackson Immuno Research laboratories, Inc.	Cat# 706-605-148
Donkey anti-Rabbit IgG (H+L) Highly CrossAdsorbed Secondary Antibody, Alexa Fluor 594 (1:400)	ThermoFisher Scientific	Cat# A32754
Donkey anti-Mouse IgG (H+L) Highly CrossAdsorbed Secondary Antibody, Alexa Fluor 594 (1:400)	ThermoFisher Scientific	Cat# A32744
Donkey anti-Goat IgG (H+L) Highly CrossAdsorbed Secondary Antibody, Alexa Fluor 488 (1:400)	ThermoFisher Scientific	Cat# A32814
Donkey anti-Rabbit IgG (H+L) Highly CrossAdsorbed Secondary Antibody, Alexa Fluor 488 (1:400)	ThermoFisher Scientific	Cat# A32790
Donkey anti-Mouse IgG (H+L) Secondary Antibody, Alexa Fluor 488 (1:400)	ThermoFisher Scientific	Cat# A21202
**Bacterial and virus strains**		
SARS-CoV-2 Virus; Variant: 2019-nCoV/USA-WA1/2020	https://www.ncbi.nlm.nih.gov/nuccore	Accession number MN985325.1
**Biological samples**		
Human Pancreas Tissue Biopsy (control)	Prodo Labs, Aliso Viejo, CA	https://prodolabs.com/
Non-human Primate Pancreas Tissue Sections (infected and control)	Tulane National Primate Research Center	https://tnprc.tulane.edu/
Human Pancreas Tissue Biopsy (infected)	Louisianna State University Health Sciences Center School of Medicine	https://www.medschool.lsuhsc.edu/pathology/
**Chemicals, peptides, and recombinant proteins**		
DPBS	Thermofisher Scientific	Cat# 21600010
Antigen Decloaker, 10X	BioCare Medical	Cat # CB910M
DAPI (4',6-Diamidino-2-Phenylindole, Dihydrochloride)	Thermofisher Scientific	Cat# 1306
Glutaraldehyde 25% EM grade	Ted Pella, Inc	Cat# 18426
Paraformaldehyde 16%	Ted Pella, Inc	Cat# 18505
Osmium Tetroxide 4%	Polysciences Inc.	Cat# 0972A
Uranyl Acetate	Ted Pella, Inc	Cat# 19481
Eponate 12 Resin Kit	Ted Pella, Inc	Cat# 18012
Antigen Decloaker, 10X	BioCare Medical	Cat # CB910M
Protein Block, Serum-Free	Dako/ Agilent	Cat # X090930-2
ImmEdge Pen	Vector Laboratories	Cat# H-4000
Normal Donkey Serum	Jackson Immuno Research laboratories, Inc.	Cat# 017-000-121
ProLong™ Diamond Antifade Mountant	ThermoFisher Scientific	Cat# P36965
**Critical commercial assays**		
Monkey Pancreatic Lipase ELISA Kit; 96-Strip-Wells	MYBIOSOURCE LLC	MBS006740

Monkey Amylase Alpha 2, Pancreatic ELISA Kit; 96-Strip-Wells	MYBIOSOURCE LLC	MBS750403
Vector® TrueVIEW® Autofluorescence Quenching Kit	Vector Laboratories	SP-8400-15
**Deposited data**		
Human reference genome NCBI build 38, GRCh38	Genome Reference Consortium	https://www.ncbi.nlm.nih.gov/assembly/GCF_000001405.39
Single nucleus and in situ RNA sequencing reveals cell topographies in the human pancreas (EGAS00001004653)	European Genome-Phenome Archive	https://ega-archive.org/studies/EGAS00001004653
Single-cell RNA-seq analysis of human pancreas from healthy individuals and type 2 diabetes patients (E-MTAB-5061)	EMBL-EBI Array Express	https://www.ebi.ac.uk/arrayexpress/experiments/E-MTAB-5061/
High-resolution single cell RNAseq of ALK3-expressing human pancreatic ductal cells (GSE131886)	Gene Expression Omnibus	https://www.ncbi.nlm.nih.gov/geo/query/acc.cgi?acc=GSE131886
A single-cell transcriptomic map of the human and mouse pancreas reveals inter- and intra-cell population structure (GSE84133)	Gene Expression Omnibus	https://www.ncbi.nlm.nih.gov/geo/query/acc.cgi?acc=GSE84133
A single-cell transcriptome atlas of the human pancreas [CEL-seq2] (GSE85241)	Gene Expression Omnibus	https://www.ncbi.nlm.nih.gov/geo/query/acc.cgi?acc=GSE85241
Single cell transcriptomics defines human islet cell signatures and reveals cell-type-specific expression changes in type 2 diabetes [single cell] (GSE86469)	Gene Expression Omnibus	https://www.ncbi.nlm.nih.gov/geo/query/acc.cgi?acc=GSE86469
**Experimental models: Organisms/strains**		
Non-Human Primate African Green (*Chlorocebus sabaeus*)	Tulane National Primate Research Center	https://tnprc.tulane.edu/
Non-Human Primate Rhesus macaque (*Macaca mulatta*)	Tulane National Primate Research Center	https://tnprc.tulane.edu/
**Software and algorithms**		
R v4.0.2 (64x bit, for Windows)	The R Consortium	https://cran.r-project.org/bin/windows/base/old/4.0.2/
RStudio v1.2.1335 (64x bit, for Windows)	The R Consortium	https://www.rstudio.com/products/rstudio/
GraphPad Prism v9.1.0.221	GraphPad	https://www.graphpad.com/scientific-software/prism/
Python v3.5.7	Python Software Foundation (14)	https://www.python.org/downloads/release/python-357/
Seurat v3.2.2.9002 and v4.0.1	The Satija Lab NYU Center for Genomics and Systems Biology (15, 16)	https://satijalab.org/seurat/
Fiji ImageJ	Dev: Schindelin , Eliceiri/LOCI, Tomancak, Jag, Saalfeld and other labs worldwide (11)	https://imagej.net/Fiji
Coloc2	Dev: Schindelin, White and Kazimiers Labs (11)	https://imagej.net/Coloc_2
EzColocalization	The Lim Lab (12)	https://github.com/DrHanLim/EzColocalization
NIS-Elements Confocal	Nikon	https://www.microscope.healthcare.nikon.com/en_EU/products/software/nis-elements
Zeiss Zen 3.3 blue	Zeiss	https://www.zeiss.com/microscopy/us/products/imaging-systems/axioscan-for-biology.html#applications
AMT Image Capture Engine V602 software	AMT	https://amtimaging.com/
CentOS 6.5 (64x bit, for Windows)	Linux	https://wiki.centos.org/Download

Windows 10 Professional (64x bit)	Microsoft	https://www.microsoft.com/en-us/p/windows-10-pro/df77x4d43rkt?activetab=pivot%3aoverviewtab

Supplemental references:

1. Council NR. Guide for the care and use of laboratory animals. 2010.
2. Blair RV, Vaccari M, Doyle-Meyers LA, Roy CJ, Russell-Lodrigue K, Fahlberg M, et al. Acute Respiratory Distress in Aged, SARS-CoV-2-Infected African Green Monkeys but Not Rhesus Macaques. *The American journal of pathology.* 2021;191(2):274-82.
3. Harcourt J, Tamin A, Lu X, Kamili S, Sakthivel SK, Murray J, et al. Severe acute respiratory syndrome coronavirus 2 from patient with coronavirus disease, United States. *Emerging infectious diseases.* 2020;26(6):1266.
4. Lawlor N, George J, Bolisetty M, Kursawe R, Sun L, Sivakamasundari V, et al. Single-cell transcriptomes identify human islet cell signatures and reveal cell-type-specific expression changes in type 2 diabetes. *Genome research.* 2017;27(2):208-22.
5. Muraro MJ, Dharmadhikari G, Grün D, Groen N, Dielen T, Jansen E, et al. A Single-Cell Transcriptome Atlas of the Human Pancreas. *Cell systems.* 2016;3(4):385-94.e3.
6. Baron M, Veres A, Wolock SL, Faust AL, Gaujoux R, Vetere A, et al. A Single-Cell Transcriptomic Map of the Human and Mouse Pancreas Reveals Inter- and Intra-cell Population Structure. *Cell systems.* 2016;3(4):346-60.e4.
7. Segerstolpe Å, Palasantza A, Eliasson P, Andersson EM, Andréasson AC, Sun X, et al. Single-Cell Transcriptome Profiling of Human Pancreatic Islets in Health and Type 2 Diabetes. *Cell metabolism.* 2016;24(4):593-607.
8. Qadir MMF, Álvarez-Cubela S, Klein D, van Dijk J, Muñiz-Anquela R, Moreno-Hernández YB, et al. Single-cell resolution analysis of the human pancreatic ductal progenitor cell niche. *Proceedings of the National Academy of Sciences.* 2020;117(20):10876.

9. Tosti L, Hang Y, Debnath O, Tiesmeyer S, Trefzer T, Steiger K, et al. Single-Nucleus and In Situ RNA-Sequencing Reveal Cell Topographies in the Human Pancreas. *Gastroenterology.* 2020.

10. Qadir MMF, Álvarez-Cubela S, Weitz J, Panzer JK, Klein D, Moreno-Hernández Y, et al. Long-term culture of human pancreatic slices as a model to study real-time islet regeneration. *Nature communications.* 2020;11(1):3265.

11. Schindelin J, Arganda-Carreras I, Frise E, Kaynig V, Longair M, Pietzsch T, et al. Fiji: an open-source platform for biological-image analysis. *Nature Methods.* 2012;9(7):676-82.

12. Stauffer W, Sheng H, and Lim HN. EzColocalization: An ImageJ plugin for visualizing and measuring colocalization in cells and organisms. *Scientific Reports.* 2018;8(1):15764.

13. Hadi AM, Mouchaers KTB, Schalij I, Grunberg K, Meijer GA, Vonk-Noordegraaf A, et al. Rapid quantification of myocardial fibrosis: a new macro-based automated analysis. *Cell Oncol (Dordr).* 2011;34(4):343-54.

14. Sanner MF. Python: a programming language for software integration and development. *Journal of molecular graphics & modelling.* 1999;17(1):57-61.

15. Stuart T, Butler A, Hoffman P, Hafemeister C, Papalexi E, Mauck III WM, et al. Comprehensive integration of single-cell data. *Cell.* 2019;177(7):1888-902. e21.

16. Hao Y, Hao S, Andersen-Nissen E, Mauck WM, Zheng S, Butler A, et al. Integrated analysis of multimodal single-cell data. *bioRxiv.* 2020.
